# Evans Syndrome Complicated by Intratubular Hemoglobin Cast Nephropathy

**DOI:** 10.1155/2017/5184587

**Published:** 2017-10-15

**Authors:** Iván González, Rehan Rais, Joseph P. Gaut, Louis P. Dehner

**Affiliations:** The Lauren V. Ackerman Laboratory of Surgical Pathology, St. Louis Children's Hospital, Washington University Medical Center, St. Louis, MO, USA

## Abstract

Evans syndrome (ES) is a rare autoimmune disorder whose exact pathophysiology is unknown. It is characterized by the simultaneous or subsequent development of autoimmune hemolytic anemia (AIHA) and immune thrombocytopenia (ITP). Intravascular hemolysis, with hemoglobinemia, is known to produce acute kidney injury; however, the development of intratubular hemoglobin casts (hemoglobin cast nephropathy) in the setting of acute hemolysis is uncommon. Likewise, the association of ES and acute renal failure is equally uncommon. We present a case of a 7-year-old girl with ES who developed acute kidney injury in the setting of intravascular hemolysis and had widespread intratubular hemoglobin casts.

## 1. Introduction

Evans syndrome (ES) is a rare autoimmune disorder first described in 1951 whose exact pathophysiology is unknown [1, 2]. It is characterized by the simultaneous or subsequent development of autoimmune hemolytic anemia (AIHA) and immune thrombocytopenia (ITP); it is classified as primary or secondary depending upon its association with others diseases, such as systemic lupus erythematous (SLE), primary antiphospholipid syndrome, Sjögren syndrome, IgA deficiency, Hodgkin lymphoma, and chronic lymphocytic leukemia [1–5].

Intravascular hemolysis, with hemoglobinemia, is known to produce acute kidney injury; however, the development of intratubular hemoglobin casts (hemoglobin cast nephropathy) in the setting of acute hemolysis is uncommon as evidenced by its documentation by the rare individual case report [3]. Likewise, the association of ES and acute renal failure is equally uncommon, in contrast to other hematologic disorders such as sickle cell disease [4–6]. We present a case of a 7-year-old girl with ES who developed acute kidney injury in the setting of intravascular hemolysis and had widespread intratubular hemoglobin casts which was documented at the postmortem examination.

## 2. Case Report

### 2.1. Clinical History

The patient was a 7-year-old girl with a past medical history of an unbalanced atrioventricular canal defect with aortic hypoplasia and coarctation which was status after Norwood procedure and ES. The patient's initial presentation of ES occurred at 6 years of age with a Coombs positive hemolytic anemia with a hemoglobin level of 6.6 g/dl (reference value: 11.5–15.5 g/dl) and a total bilirubin of 12 mg/dl (reference value: 0.0–1.2 mg/dl), with a direct bilirubin level of 1.9 mg/dl (reference value: 0.0–0.4 mg/dl). An accompanying neutropenia was also detected presumably secondary to a viral illness until the detection of granulocyte antibodies and there were also low T-lymphocyte levels with a normal CD4/CD8 ratio.

Two months prior to death, she presented to the emergency department (ED) with fever and generalized abdominal pain over the prior 5 days, after reported exposure to a family member with symptoms of an upper respiratory infection. She was found to be anemic, leukopenic, and thrombocytopenic as manifestations of an exacerbation of the previously diagnosed ES with autoimmune granulocytopenia. Serum creatinine level was 1.7 mg/dl (reference range: 0.2–0.8 mg/dl) and blood urea nitrogen (BUN) level was 45 mg/dl (reference range: 9–18 mg/dl). A kidney biopsy revealed acute tubulointerstitial nephritis with acute tubular injury, consistent with a drug reaction or infection. She was treated with steroids and the serum creatinine level decreased to baseline levels (0.8–1.0 mg/dl).

One month later, she again presented to the ED with generalized abdominal pain and distention for one week. Facial edema was noted on physical examination. At this time there was no evidence of anemia, thrombocytopenia, or leukopenia. Serum creatinine level was 0.8 mg/dl (reference value: 0.2–0.8 mg/dl) and serum BUN level was 31 mg/dl (reference value: 9–18 mg/dl). The serum brain natriuretic peptide (BNP) level was elevated at 7,534 pg/ml (reference value: 0–39 pg/ml). Cardiac catheterization was performed showing aorta coarctation and multiple vascular collaterals in the lung. The day after the diagnostic catheterization the serum creatinine level increased to 1.2 mg/dl and serum BUN level increased to 56 mg/dl.

One week later the patient developed a Coombs negative hemolytic anemia with hemoglobin level of 6.3 g/dl (reference value: 11.5–15.5 g/dl), with a serum lactate dehydrogenase level of >2500 Units/L (reference value: 100–300 Units/L); leukocytes and platelets were within the references values. Total serum bilirubin level was 35 mg/dl (reference value: 0.0–1.2 mg/dl), with a direct bilirubin level of 28.4 mg/dl (reference value: 0.0–0.4 mg/dl). Urine analysis showed the presence of *β*eta-2 microglobulin with a level of 5.5 mg/L (reference value: 0.0–0.2 mg/L). Liver chemistry studies showed an elevated ALT level of 88 Units/L (reference value: 10–40 Units/L) and AST level of 480 Units/L (reference value: 10–60 Units/L). The patient required daily red blood cells transfusions, with a total of 11 units of RBCs over 10 days. Her renal function continued to deteriorate with an accompanying decline in mental status and severe metabolic acidosis with a pH of 7.04 (reference value: 7.35–7.45). She subsequently died and an autopsy was performed.

### 2.2. Pathologic Findings

**Figure 1:**
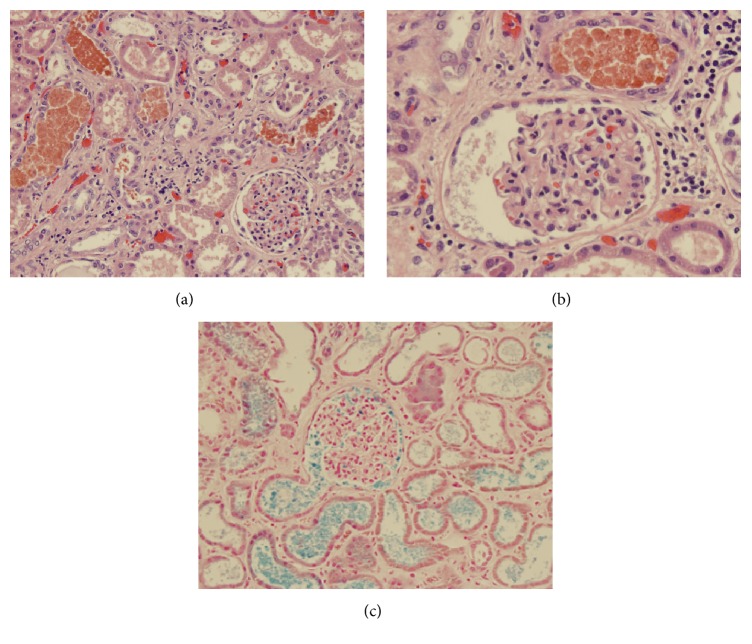
((a), (b)) Unremarkable glomeruli with acute tubular injury with scattered inflammatory cells and intratubular granular-pigmented cast and (c) Prussian blue stain highlighting the intratubular and intraglomerular pigmented cast.

The kidneys at autopsy showed proximal tubular dilatation with flattened epithelium; many of the dilated tubules contained reddish casts (Figures 1(a) and 1(b)). These casts stained positively for iron with the Prussian blue stain (Figure 1(c)). Unlike chronic hemolysis, the tubular epithelial cells failed to demonstrate any iron deposition in the cytoplasm.

The remainder of the postmortem examination revealed cardiomegaly (337 gm) with right ventricular hypertrophy, AV canal defect, and hypoplastic aortic arch. The liver showed advanced fibrosis with features of cardiac cirrhosis and splenomegaly with extramedullary hematopoiesis.

## 3. Discussion

The current study presents the case of a 7-year-old child who developed ES, an autoimmune multilineage cytopathy, which is characterized by autoimmune hemolytic anemia (AIHA) and immune thrombocytopenic purpura (ITP) [7]. Though the patient did not have a diagnosable underlying autoimmune disorder such as systemic lupus erythematosus (SLE), the family history was remarkable for SLE, Sjögren syndrome, and Hodgkin lymphoma. Anti-granulocyte antibodies were detected as well as low T-lymphocyte counts, but the CD4/CD8 ratio was within the normal range. There was no definable primary immunodeficiency disorder.

The relationship between ES and renal disease is mainly through those uncommon cases with associated SLE and lupus nephritis [8]. Another case of acute renal failure and ES was reported in a patient who developed a polyclonal lymphoproliferative disorder resulting in diffuse lymphadenopathy and renomegaly with infiltration of kidneys by lymphoid cells; there was eventual clinical resolution and the pathogenesis was thought to be on the basis of drug or toxic reaction [4]. There is otherwise no known relationship between ES and acute or chronic renal failure.

Our case of ES presents another avenue of acute renal failure, the development of hemoglobin casts in the kidney, which was suspected before death and confirmed at autopsy. It is interesting that ES is not more often complicated by the formation of hemoglobin casts since intravascular hemolysis is a well-documented cause of acute or chronic renal failure; one important example of the latter is sickle cell disease with vasoocclusion and hemolysis [5, 6]. However, hemoglobin casts as a primary cause of acute renal failure remain extremely uncommon [3]. A similar process is myoglobin cast nephropathy [9].

Interest in the role of free hemoglobin in renal injury is well-documented in experimental and clinical studies dating over the past 50 or more years [10–13]. With intravascular hemolysis, free hemoglobin becomes bound to haptoglobin forming a hemoglobin-haptoglobin complex which inhibits hemoglobin filtration and prevents the toxic effects on the tubular epithelium [14]. In those cases of severe hemolysis haptoglobin becomes saturated and free hemoglobin or its iron-containing tetrapyrrole is filtrated through the glomerulus where its toxic effects include instability of tubular epithelium membranes, activation of caspases/cathepsins, denaturation of DNA, and mitochondrial injury as a summary of a complex pathogenesis [3, 15]. The pathologic consequence is hemoglobin cast nephropathy which in our case led to acute renal failure and death.
